# *Bacillus/Trapa japonica Fruit Extract Ferment Filtrate* enhances human hair follicle dermal papilla cell proliferation via the Akt/ERK/GSK-3β signaling pathway

**DOI:** 10.1186/s12906-019-2514-8

**Published:** 2019-05-14

**Authors:** Gun-He Nam, Kyung-Jo Jo, Ye-Seul Park, Hye Won Kawk, Je-Geun Yoo, Jin Dong Jang, Sang Moon Kang, Sang-Yong Kim, Young-Min Kim

**Affiliations:** 1 Department of Biological science and Biotechnology, College of Life science and Nano technology, Hannam University, 1646 Yuseong-daero, Yuseong-gu, Daejeon, 34054 South Korea; 2 Department of Food Science & Bio Technology, Shinansan University, Daehakro Danwon-gu, Ansan City, Gyenggi-do South Korea; 3 Doori Cosmetics Co.,Ltd., 11F Galaxy Tower, 175, Saimdang-ro, Seocho-gu, Seoul, South Korea; 4 ANPEP INC., 13, Oksansandan 1-ro, Oksan-myeon, Heungdeok-gu, Cheongju-si, Chungcheongbuk-do Republic of Korea

## Abstract

**Background:**

Despite advances in medical treatments, the proportion of the population suffering from alopecia is increasing, creating a need for new treatments to control hair loss and prevent balding. Treatments based on plant-derived compounds could potentially prevent hair loss. Human hair follicle dermal papilla (HDP) cells, a type of specialized fibroblast in the hair bulb, play an essential role in controlling hair growth and in conditions such as androgenic alopecia. We examined the effect of *Bacillus/Trapa japonica* fruit ferment filtrate extracts (TJFs) on HDP cells to determine whether activation of the Akt/ERK/GSK-3β signaling pathway improved HDP cell proliferation.

**Methods:**

We prepared TJFs using various methods. The extract properties were analyzed using WST-1, Lowry, and cell migration assays as well as immunofluorescence staining. We also determined the cell cycle stage and performed western blotting and an *in ovo* chick chorioallantoic membrane assay. Last, we constructed an organotypic three-dimensional cell culture model for immunohistochemical use.

**Results:**

Our study confirmed that the TJFs contained numerous peptides and five unknown fractions. The TJFs stimulated HDP cell proliferation and migration via the Akt/ERK/GSK-3β signaling pathway. To verify that the Akt/ERK/GSK-3β pathway affected HDP cell proliferation, we treated HDP cells with LY294002 (an Akt inhibitor), BIO (a GSK-3β inhibitor), and PD98059 (an ERK inhibitor). The TJFs also induced cell cycle progression, inhibited type І 5α-reductase, decreased apoptosis, and enhanced angiogenesis (vascular expansion). In addition to these signaling pathways, proteins including insulin-like growth factor-1 and keratinocyte growth factor, stimulating hair growth, were detected in the three-dimensional cell culture model.

**Conclusions:**

Our results confirmed that TJFs enhance HDP cell proliferation via the Akt/ERK/GSK-3β signaling pathway, suggesting a potential treatment for alopecia.

## Background

The independent life cycle of each hair follicle is divided into three phases: growth (anagen), regression (catagen), and resting (telogen) [1, 2]. Each stage has distinct morphological features, such as cell proliferation and differentiation, hair growth, and elimination. Human hair follicle dermal papilla (HDP) cells, a type of specialized fibroblast cell in the hair bulb, play an essential role in controlling hair growth and in conditions such as androgenic alopecia [3, 4]. Therefore, factors affecting HDP function are important targets for ameliorating alopecia. To date, the mechanism underlying the regulation of hair growth by HDP cells remains unclear. Various signal proteins and growth factors are involved in controlling the hair growth cycle. According to previous alopecia studies, among the signal proteins present in the skin, keratinocyte growth factor (KGF) and insulin-like growth factor-1 (IGF-1) affect hair growth [5–8]. The male hormone progesterone shortens the hair growth stages, inducing the hair to reach the regression phase faster than usual. Type I 5α-reductase, participating in male hormone metabolism in HDP cells, has been linked to alopecia [9, 10]. Additionally, environmental stress increases apoptosis, a process whereby cells die in response to self-generated signals, in HDP cells. Excessive apoptosis in HDP cells increases the rate of hair loss [11, 12]. Reducing HDP apoptosis and inhibiting the production of type I 5α-reductase is effective in preventing alopecia, and inducing HDP growth is critical for its treatment. Several signaling pathways are associated with cell proliferation. The Akt/ERK/GSK-3β pathway in HDP cells promotes hair growth [13–16]. Akt, a serine/threonine kinase, is a critical protein that acts downstream of the P13K pathway to modulate the response of cells to external stimuli and regulate cell proliferation and survival by controlling various internal signals [17]. In the normal physiological state, Akt is activated by growth factors and genes involved in regulating a complex network in HDP cells. Phosphorylated Akt exists in various organelles, including mitochondria and nuclei, where it interacts with other molecules. The Wnt pathway is a cellular signal transmission pathway that determines cell fate and controls cell proliferation and differentiation. A significant protein in the Wnt pathway, GSK-3β, blocks β-catenin breakdown, and β-catenin deposited in the cytoplasm enters the nucleus and transcribes various genes necessary for cell growth and survival [18, 19]. The ERK signaling pathway controls the cell cycle of proliferation, differentiation, and apoptosis [20]. The growth of HDP cells via vascular expansion and hair via potassium channels is promoted through the Akt/ERK/GSK-3β signaling pathway [21].

The modern stressful living environment has increased the incidence of alopecia, and the growing interest in skin care has fueled the social attention on hair loss treatment [22]. The only drug approved by the Food and Drug Administration for alopecia treatment is minoxidil; therefore, it is crucial to identify new drugs that could promote hair growth. A recent study has demonstrated that ingredients derived from natural substances prevent hair loss, are usually safe, and are highly likely to be successful because of their short development period [23, 24]. The global market for natural products is growing, and research on substances derived from natural sources is a high-value industry with high investment efficiency worldwide [25]. Because of the difficulty in developing new medicines through chemical and pharmaceutical approaches, chemical and biopharmaceutical companies are increasingly focusing on natural products for drug development.

As one of the methods to develop new medicinal materials, fermentation is a process that breaks down natural products through the catalytic decomposition by microorganisms at a constant temperature and humidity after a specific treatment. This process is performed under strict temperature and humidity conditions via different methods depending on the type of natural products. Through fermentation, unabsorbed glycosides in the intestines can be bioconverted into non-glycosides, and this increases the absorption and bioavailability of the pharmaceutical ingredients in the body. Our study focuses on *Trapa japonica*, a floating aquatic plant found in ponds or other slow-moving water bodies, used as a traditional food as well as a tonic in East Asia. Previous studies have revealed that *T. japonica* exerts antioxidant and anticancer effects; however, the components of *T. japonica* remain unknown [26]. Based on studies where effective substances were extracted from a traditional plant, we used a water fermentation process to develop new functional materials, which maximized the physiological activity of *T. japonica* through interactions between natural substances and microorganisms. Thereafter, solvents were used to separate the fractions [27, 28]. To reinforce the composition of the natural products, we used two types of mixed microorganisms. We investigated the effect of *Trapa japonica* fruit ferment filtrate extracts (TJFs) on HDP cells both in vitro and ex vivo to identify and isolate new substances for alopecia treatment.

## Methods

### Preparation of Bacillus/Trapa japonica fruit extract ferment filtrate (TJFs)

*T. japonica* (Texa and representative voucher specimen number: KP255650) *was* purchased from BS corporation (BS corporation, KOREA). It was grown in China. The hot water extraction of *T. japonica* was heated in a 37 °C by adding *T. japonica* and distilled water, then filtered out the vacuum and concentrated in the water bath. Then, the hot water extraction of *T. japonica* was fermented using two species of microorganisms: *Bacillus methulotrophicus* and *Bacillus subtilis*. *T. japonica* and medium were mixed with glucose, yeast extract and soytone. The final fermentation was performed by mixing microorganisms. It was fermented 72 h in a 37 °C fermentor (Fermentec, KOREA). After fermentation was completed, it was centrifuged and filtered with a 0.2 μm filter to completely remove the microorganisms. The fermented *T. japonica* extract was sequentially separated into Hexane, CH2Cl2, EtOAc, N-BuOH and water layers. Fractions were run in the order Hexane, CH2Cl2, EtOAc, and N-BuOH. Afterwards, the water layer was separated by using the Silica gel column (Waters, USA) was performed to divide into six fractions (fraction 1 ~ 6). Afterwards Sehpadex-LH20 column (Waters, USA) was performed to divide into six fractions. Compound 1–6 was then obtained via HPLC prep HPLC Xterra C18 ODS column (Waters, USA). fraction was refined using HPLC (Waters, USA). The mobile phase consisted of distilled water: ACN: MeOH solution (90:5:5, v/v) pumped at a rate of 1 ml/min. The combined filtrate was concentrated under vacuum, and completely dried by freeze drying. TJFs (consisting of 6 compounds) powder was dissolved in distilled water. GH Nam and SM Kang undertook the identification of TJFs in this study.

### Lowry assay

The amount of peptide in TJFs were measured using a lowry assay. It were Calculated by a standard curve using Bovine serum albumin (Sigma, USA). Lowry solution consists of a mixture of solution A and B (Solution A: 4 mg/mL NaOH and 20 mg/mL Na2CO3 in water Added 2 g of NaOH and 10 g of Na2CO3 to 400 mL water while stirring until completely dissolved, then adjust volume to 500 mL. Solution B: 10 mg/mL Potassium Sodium Tartrate and 5 mg/ mL CuSO4 in water). Add 100 mg Potassium Sodium Tartrate and 50 mg CuSO4 (cupric sulfate to 8 mL of water in a tube. Shake the mixture until solids are completely dissolved, adjust volume to 10 mL (50:1 mix of solutions A and B). Prepare samples by adding 2, 5, and 10 μL of sample into a glass tube and adjust total volume to 200 μL. To each tube added 1 mL Lowry’s Solution, vortex, wait for 15 mins. To each tube added 100 μL 1.0 N Folin’s Phenol reagent (Sigma, USA) while vortexing, wait for 30 mins. Absorbance is measured at 750 nm.

### Reagent

Wst-1 assay kit was purchased from Daeillab (Daeillab, Korea). LY294002 (PI3K/Akt inhibitor) and PD98059 (Erk inhibitor), L-Ascorbic acid were purchased from Sigma Aldrich (Sigma Aldrich, USA). BIO (GSK-3β inhibitor) was purchased from Santa Cruz Biotechnology (Santa Cruz Biotechnology, USA). Specific antibodies such as p-Akt, total-Akt, β-actin, Cyclin E, p-cdk2, p21 were obtained from Cell Signaling Technology (Beverly, USA). and p-GSK-3β, total-GSK-3β, p-Erk, total-Erk, Active-caspase-3 antibodies were purchased from Abcam (Cambridge, USA). Muse™ Muse™ Cell Cycle Kit (MCH100106) and Muse™ Cell Analyzer (PB4455ENEU) were purchased from Millipore (EMD Millipore Corporation, Germany). 3M™ Tegaderm (sterile barrier to external contaminants) was purchased from 3 M (3 M, USA).

### Cell culture

Human hair Follicle dermal papilla cells were obtained from CEFO (CEFO, KOREA). Human hair Follicle dermal papilla cells were grown in DMEM medium (Hyclone, USA) containing 10% Fetal bovine serum (Hyclone, USA) and 1% antibiotics (100 mg/streptomycin, 100 U/ml penicillin) at 37 °C in a 5% CO_2_ atmosphere.

### Wst-1 assay

Cells were seeded at 3.8 **×** 10^5^ cells/ml in a 12-well plate for 24 h and were incubated with TJFs (0.1 and 1%) for 24–48 h. Certain inhibitors (LY294002, BIO, PD98059) were treated for 24 h. Following incubation with the TJFs, the cells were incubated with a 100 μl/ml Wst-1 solution (Daeillab, Korea) for 60 min. Then, the optical densities of the solutions were quantified at a 450 nm wavelength by using a FLUOstar Omega (BMG labtech, Germany).

### Cell migration assay

Seed the cells in a 6-well plate and culture until confluent. Then, using a 200 μl pipette tip (CORNING, USA) make a straight scratch, simulating a wound and were incubated with various concentrations of TJFs (0.1 and 1%) for 24 h. The wound healing area was photographed under a microscope (Carl Zeiss, USA). The photographs were taken at a magnification of × 200.

### Immunofluorescence (IF) staining

Cells were seeded at 1 **×** 10^5^ cells/ml in a 12-well plate with cover glasses and incubated for 24 h. Following treatment with TJFs (1%), The cells were stained with 0.7 μM Hoechst 33342 for 10 min and fixed with 10% Neutral buffered formalin for 20 min. Then the cells were washed with PBS twice, Cells were permeabilized with 100% MeOH and blocking in 10% normal goat serum. Cells were then incubated overnight at 4 °C with active-caspase 3 primary antibody. On the second day, cells were washed with PBS and incubated with a secondary antibody for 1 h. The coverslips were mounted for fluorescence microscope observation. Subsequently, the cells were observed using a confocal microscope (Olympus, Japan).

### Determination of cell cycle

Cells were seeded at 9.5 **×** 10^5^ cells/ml in a 6-well plate. After 24 h incubation, cells were treated with various concentrations of TJFs (0.1 and 1%) for 24 h. Following incubation with TJFs, the cells were resuspended with PBS. And slowly add 0.2 ml of 70% ethanol. After incubate for at least 3 h at − 20 °C, the fixed cells were analyzed in Muse™ Cell Analyzer (Merck Millipore Co.).

### ELISA assay

Cells were seeded at 1 **×** 10^5^ cells/ml in a 24-well plate for 24 h transfer serum-free media and were incubated with various concentrations of TJFs (0.1 and 1%) for 24 h. Following incubation with the TJFs, incubated with SRD5A1 covered 96-well microplate at 37 °C for 60 min. Transfer 100 μl of 1X Biotinylated SRD5A1 Detector Antibody into one 96-well plate and incubate at 37 °C for 60 min. Then, add 100 μl 1X Avidin-HRP Conjugate, seal the 96-well plate (e.g. with a foil) and stand for 60 min at 37 °C. Add 90 μl of TMB Substrate into each well and incubate at 37 °C for 15–30 min. Add 50 μl of Stop Solution into each well in same order as for substrate. Tap plate gently to mix. Measure the absorbance at 450 nm with a FLUOstar Omega (BMG labtech, Germany).

### Western blotting

Cells were seeded at 1 **×** 10^6^ cells/ml in a 6-well plate. After a 24 h incubation, cells were treated with various concentrations of TJFs (0.1 and 1%) for 24 h. Certain inhibitors (LY294002, BIO, PD98059) were pre-treated for 60–120 min prior to treatment with TJFs (1%). After a 24 h, cells were rinsed twice with PBS, scraped with a lysis buffer [50 mM Tris-HCl (pH 8.0, 150 mM NaCl, 1% NP40, 0.5% sodium deoxycholate] with Halt™ Protease and Phosphatase inhibitor cocktail (ThermoFisher, USA) and subjected to the western blot analysis. Protein quantification was performed using a Bradford assay and 50 μg of protein were loaded per lane. Primary antibodies reacted overnight at 4 °C and secondary antibodies reacted for 120 min at 4 °C.

### In ovo chicken Chorioallantoic membrane (CAM) assay

Sterilize the Fertile eggs with 70% ethanol and put them in 37 °C, 45 ± 5% Humidity incubator. After 72 h incubation, drill a hole in the air sac of the Fertile egg and check the vessel. Then, put a disk containing TJFs (0.1 and 1%) and Vitamin C (0.1%) with sterilized silicon ring in the Chicken Chorioallantoic Membrane. block it with a tegarderm (sterile barrier to external contaminants) and put it back into 37 °C, 45 ± 5% Humidity incubator. After 72 h, Verify the angiogenensis effects of the TJFs through a microscope (Carl Zeiss, USA).

### Organotypic 3D cell culture model

6-well plates were pre-coated with type I collagen and incubated for 10 min in room temperature. 1 **×** 10^6^ cells/ml Human hair Follicle dermal papilla cells were seeded on 0.3-μm pore size cell culture insert plate and incubation with Matrigel and type I collagen mixture in 45 min. After cell mixture was detached from the insert plate, incubated for 3 days in the complete medium. After incubation, the 3D cell formation medium was placed in the bottom well and cultured for 2 weeks while changing the medium with TJFs (1%) every 2 days.

### Immunohistochemistry

The organotypic 3D cell cultures were fixed in 10% Neutral buffered formalin for 24–48 h, embedded in paraffin and sectioned into 5 μM thick slices. Consecutive thin cryosections (5 μM) of optimum cutting temperature compound (Sakura Finetek, USA) embedded sections were fixed in acetone at 4 °C for 10 min. Following washing in PBS, sections were treated with 3% H_2_O_2_ for 10 min to block endogenous peroxidase activity, and the sections were inhibited with 10% normal goat serum. Then, the sections were blocked and washed in PBS and incubated with a primary antibody overnight at 4 °C. On the second day, the sections were washed with PBS and incubated with a secondary antibody for 2 h, the sections were observed using a confocal microscope (Olympus, Japan).

### Statistical analysis

All the experiments were repeated at least three times and analyzed using t-tests (SPSS 20.0, USA). *p*<0.05 was considered to indicate a statistically significant difference.

## Results

### Preparation and measurement of TJFs

**Fig. 1:**
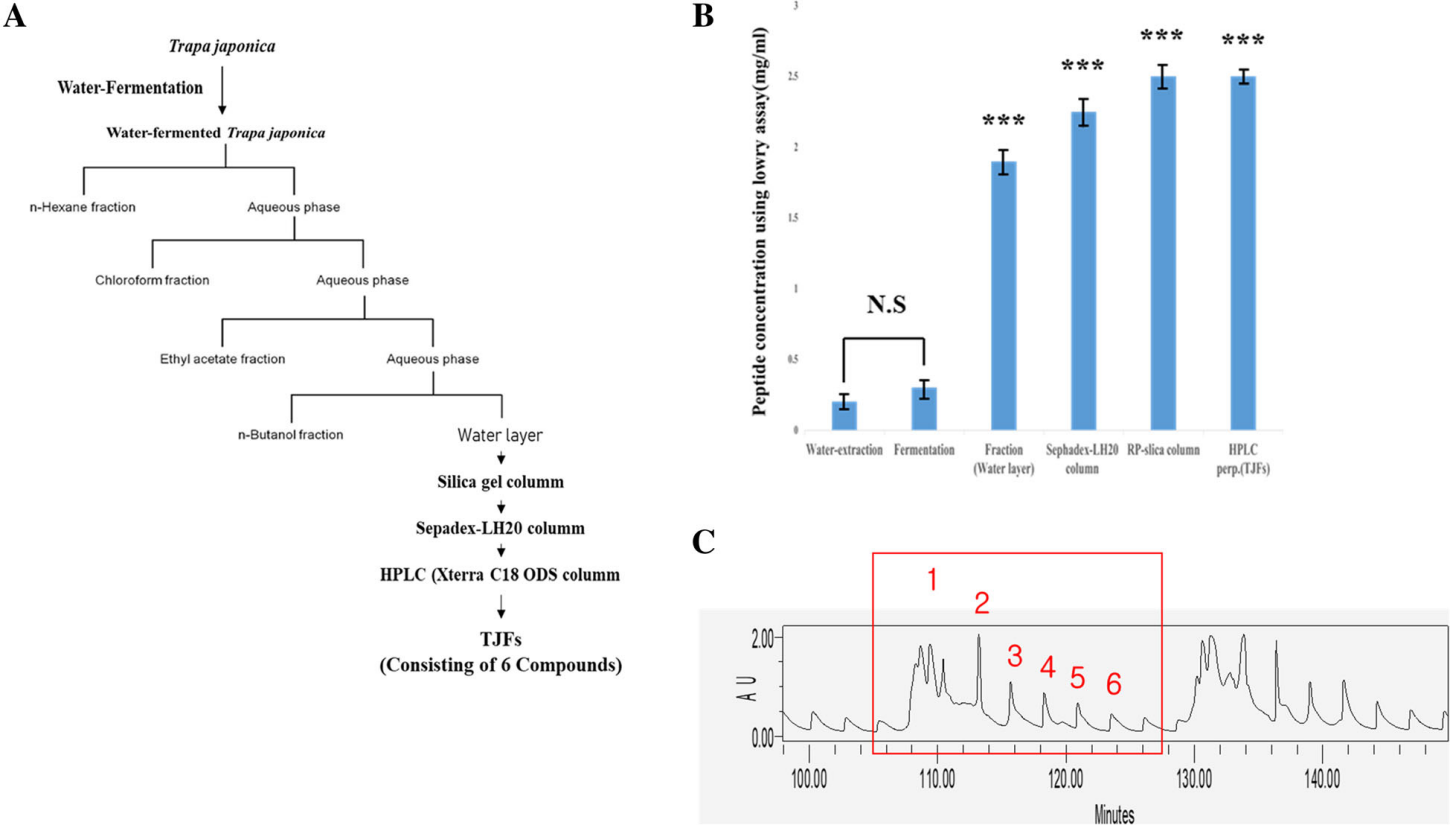
**a** Fractionation of the water-fermented *T. japonica solvent fraction* by Prep-HPLC. **b** Measurement of peptide concentration using lowry assay. **c** HPLC profile of TJFs (1–6 fractions). The statistical analysis of the data was carried out by use of an independent sample ANOVA test. *p < 0.05, **p < 0.01 and ***p < 0.001 vs. con (24 h). ^###^*p* < 0.001 (each experiment, *n* = 3). NS, not significant

In a previous study, plant fermentation produced various biologically active substances [29]. We used different solvent layers (hexane, CH_2_Cl_2_, EtOAc, n-BuOH, and water) to separate and explore the biologically active substances present in TJFs (Fig. 1a). Natural extracts typically contain a high level of glycogens, steroids, etc. However, peptides are more effective in smaller amounts than other substances. Since relatively few side effects have been associated with amino acid combinations when natural extracts have been administered to humans, we conducted experiments dependent on the peptide content. As Fig. 1b shows, the amount of peptide in the TJFs was measured using a Lowry assay. To confirm the peptide content, we isolated the proteins present in each solvent layer and quantified the different fractions using high-performance liquid chromatography (HPLC). Most peptides were isolated in the water layer. The HPLC profiles revealed the presence of six peptides (Fig. 1c).

### TJFs stimulated HDP cell proliferation and migration

**Fig. 2:**
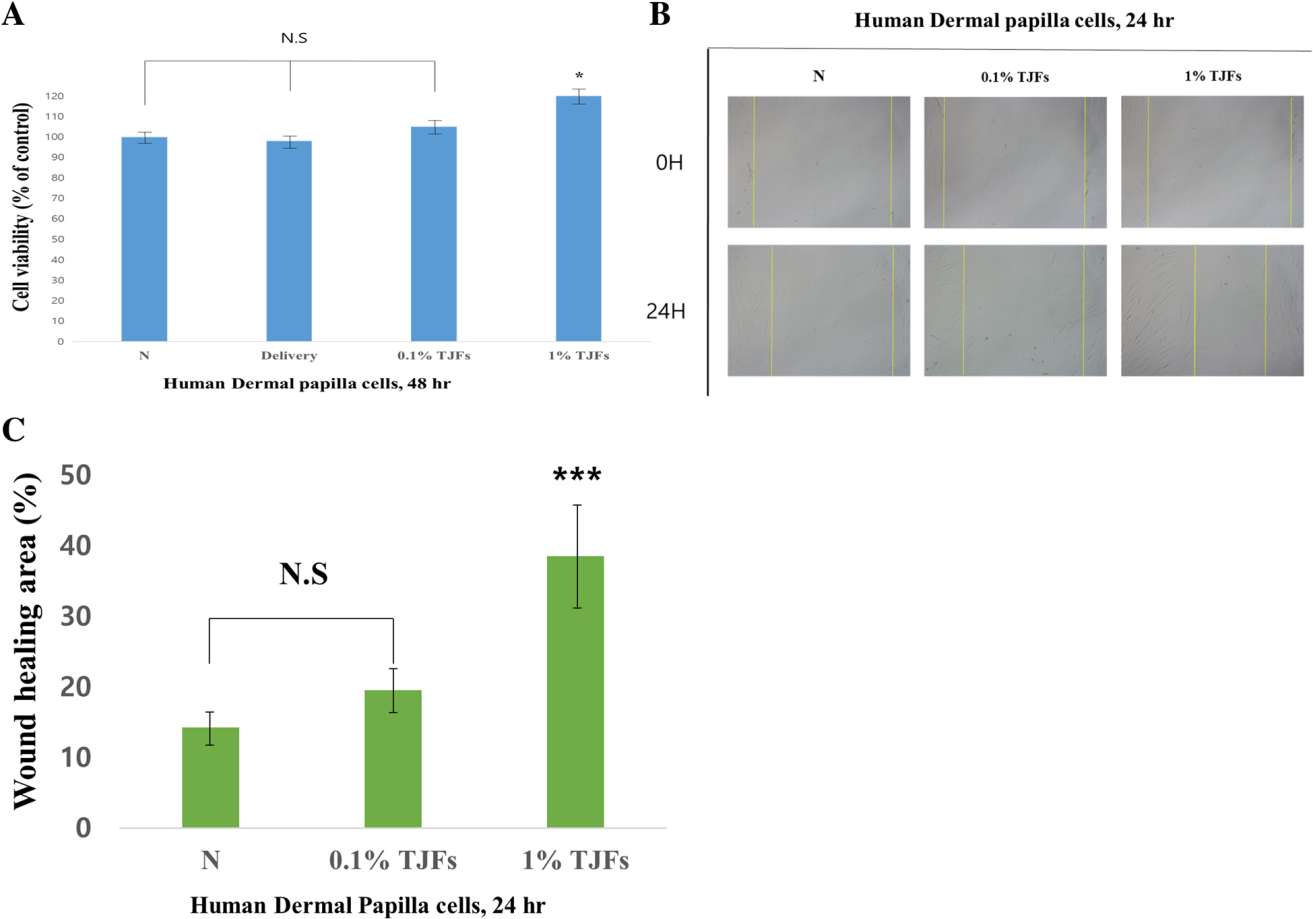
**a** Cell viability was measured by WST-1 assay. Cells were treated to with variable concentrations of TJFs (0.1 and 1%) for 48 h. **b** Cell motility was measured using a wound healing assay. Representative images of the cell migration were captured. The photographs were taken at a magnification of × 200 (**c**) The graph of wound healing area using a wound healing assay. The statistical analysis of the data was carried out by use of an independent sample ANOVA test. *p < 0.05, **p < 0.01 and ***p < 0.001 vs. con (24 h). ^###^*p* < 0.001 (each experiment, *n* = 3). NS, not significant

To test the effects of the TJFs on HDP proliferation and migration, the cells were treated with different concentrations of TJFs (0.1 and 1%) and phosphate-buffered saline in 1% dimethyl sulfoxide (delivery) and evaluated by a WST-1 and cell migration assay. The proliferation effects on HDP cells were highest at a concentration of 1% TJF, as shown in Fig. 2a. No significant toxicity was observed in the HDP cells because the viability remained > 90% when compared with the control. To determine the effect of TJFs on HDP cells, the cells were treated with different concentrations of TJFs (0.1 and 1%) and evaluated by cell migration assay. Cell migration was stimulated by the TJF treatment, as observed by a narrowing of the gap distance (Fig. 2b and c). Thus, these data demonstrated that TJFs induced HDP cell proliferation and migration.

### Cell cycle progression and inhibition of type І 5α-reductase induced by TJFs

**Fig. 3:**
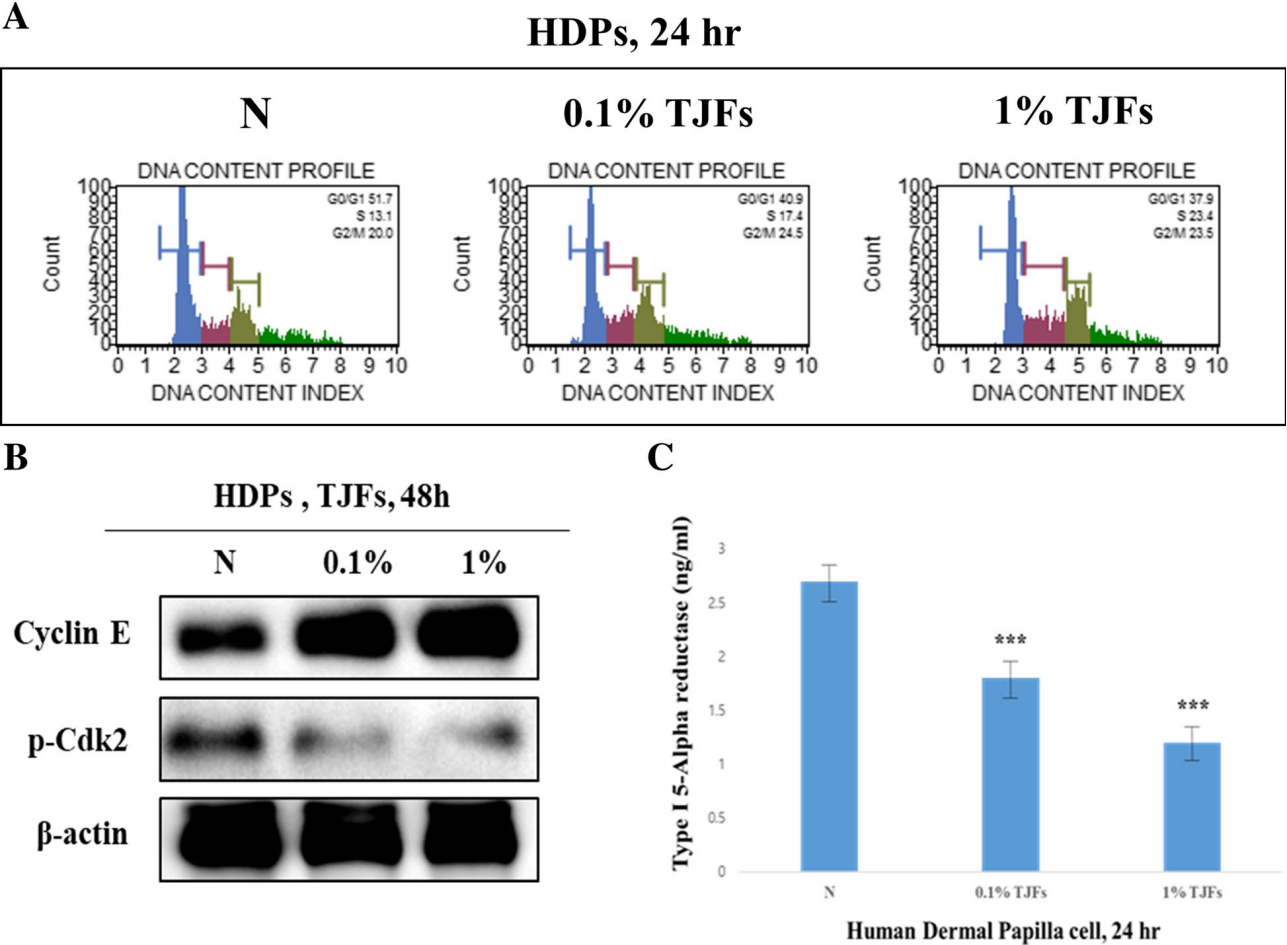
**a** TJFs occurs cell cycle progression. Cell cycle arrest effect was measured by flow cytometry. **b** Cyclin E and p-Cdk2 Protein levels were determined by Western blot analysis. The β-actin probe served as protein-loading control. **c** Measurement of Type І 5α-reductase through ELISA assay. TJFs decreased Type І 5α-reductase in HDPs. The statistical analysis of the data was carried out by use of an independent sample ANOVA test. ^*^p < 0.05, ^**^p < 0.01 and ^***^p < 0.001 vs. con (24 h). ^###^*p* < 0.001 (each experiment, *n* = 3). NS, not significant

Propidium iodide (PI) discriminates cells at different stages of the cell cycle based on differential DNA content. In the G_1_ (resting) phase, the cells contain two copies of each chromosome. As the cells begin cycling, they synthesize chromosomal DNA (S phase). The fluorescence intensity from the PI increases until all chromosomal DNA has doubled (G_2_/M phase). There was significant cell cycle progression in TJF-treated HDP cells in a dose-dependent manner, as shown in Fig. 3a and b. Following 24 h of treatment, the number of cells in the S phase was significantly elevated. In contrast, the number of cells in the G_1_ phase was significantly decreased (G_1_ phase:negative control = 51.7%, G_1_ phase + 0.1% TJF:negative control = 40.9%, G_1_ phase + 1% TJF:negative control = 37.9%). Moreover, TJF treatment also affected proteins related to cell cycle progression. Cyclin E is a member of the cyclin family and binds to G1 phase CDK2. The cyclin E/CDK2 complex is required for the transition from the G_1_ to S phase of the cell cycle, thereby initiating DNA duplication [26]. We used western blot analysis to analyze the expression of cyclin E and p-CDK2 (Fig. 3b). Our results indicated that cylin E expression significantly increased and p-CDK2 expression decreased when HDP cells were treated with the TJFs (0.1 and 1%). We performed an enzyme-linked immunosorbent assay to verify that the TJF treatment had caused a decrease in type І 5α-reductase. As Fig. 3c shows, type І 5α-reductase was decreased following treatment with 0.1 and 1% TJF compared with the negative control.

### TJFs decreased apoptosis and enhanced angiogenesis (vascular expansion)

**Fig. 4:**
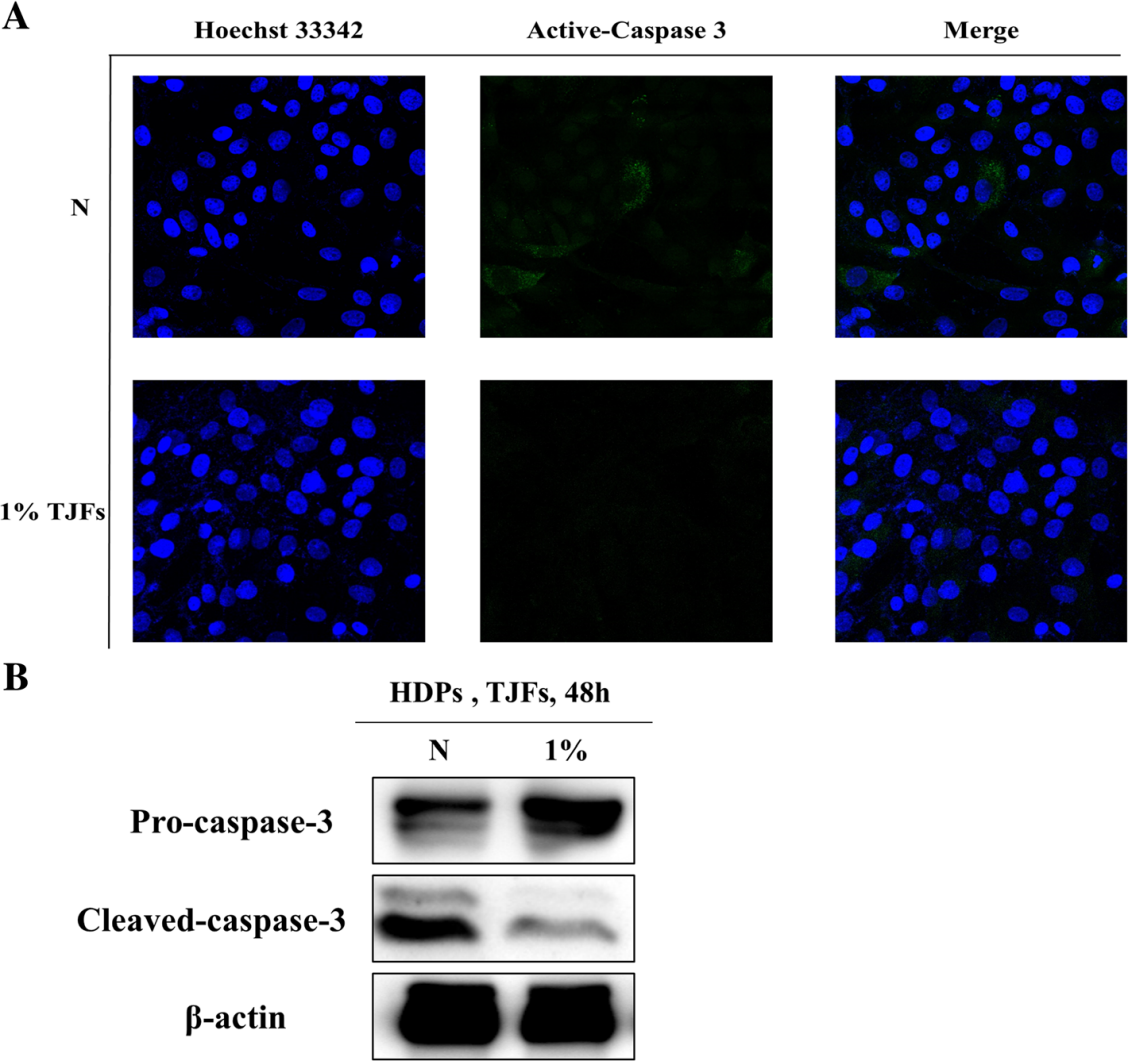
**a** Cells were treated with variable concentrations of TJFs (0.1 and 1%) for 24 h, pre-stained with Hoechst 33342 before fixation and permeabilization of cells and reacted with active-caspase 3(apoptosis inducer) primary antibody. Florescence detected by confocal microscope. **b** Pro-caspase-3 and cleaved caspase-3 Protein levels were determined by Western blot analysis. The β-actin probe served as protein-loading control

**Fig. 5:**
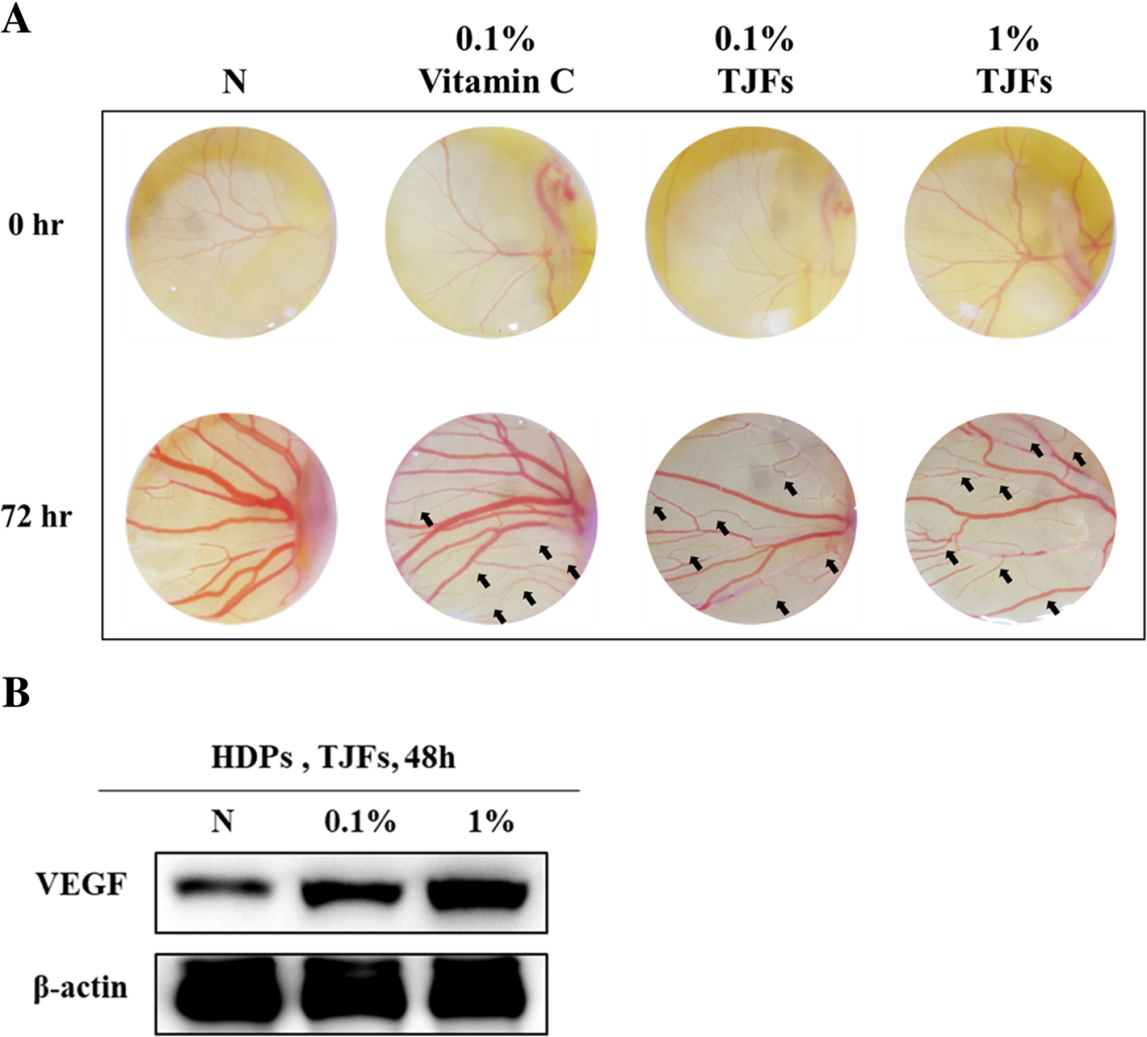
**a** Representative images of chick chorioallantoic membrane in the CAM assay are shown. The fertilized eggs were treated with or without the indicated variable concentrations of TJFs (0.1 and 1%) for 72 h. The angiogenesis effects were observed and captured (original magnification, × 10) (**b**) VEGF Protein level was determined by Western blot analysis. The β-actin probe served as protein-loading control

HDP cells require growth factors for proliferation and survival. Growth factor deprivation can lead to apoptosis, and excessive apoptosis in HDP cells leads to hair loss. Therefore, we investigated whether TJFs played a vital role in preventing apoptosis by examining caspase 3 activation as a marker of apoptosis in the presence or absence of TJFs (Fig. 4a and b). TJF-treated HDP cells demonstrated a significant decrease in the percentage of caspase 3 activation compared with the control. We also performed an *in ovo* **(**Latin for “in the egg”) chick chorioallantoic membrane (CAM) assay. As Fig. 3b shows, angiogenesis is related to vascular expansion and is essential for hair growth. To investigate the effect of TJFs on angiogenesis, TJFs (0.1 and 1%) were injected into fertile eggs and incubated for 72 h. As Fig. 5a shows, angiogenesis effects were observed with both the 0.1 and 1% TJF treatment. Because vitamin C induces angiogenesis and has a positive effect on cell growth [30, 31], we used this as a positive control. The level of angiogenesis observed in the 0.1 and 1% TJF treatment groups was comparable to that of the vitamin C treatment group. The arrows indicated the effects of angiogenesis.

Vascular endothelial growth factor (VEGF) is widely recognized as a potent angiogenic factor, and several studies have demonstrated that VEGF can promote hair growth as well as enlarge hair follicle size to result in increased hair thickness [32]. Western blot analysis revealed the expression of VEGF (Fig. 5b). Taken together, TJF treatment decreased apoptosis and enhanced angiogenesis.

### TJFs stimulated HDP cell proliferation via an Akt/ERK/GSK-3β signaling-dependent pathway

**Fig. 6:**
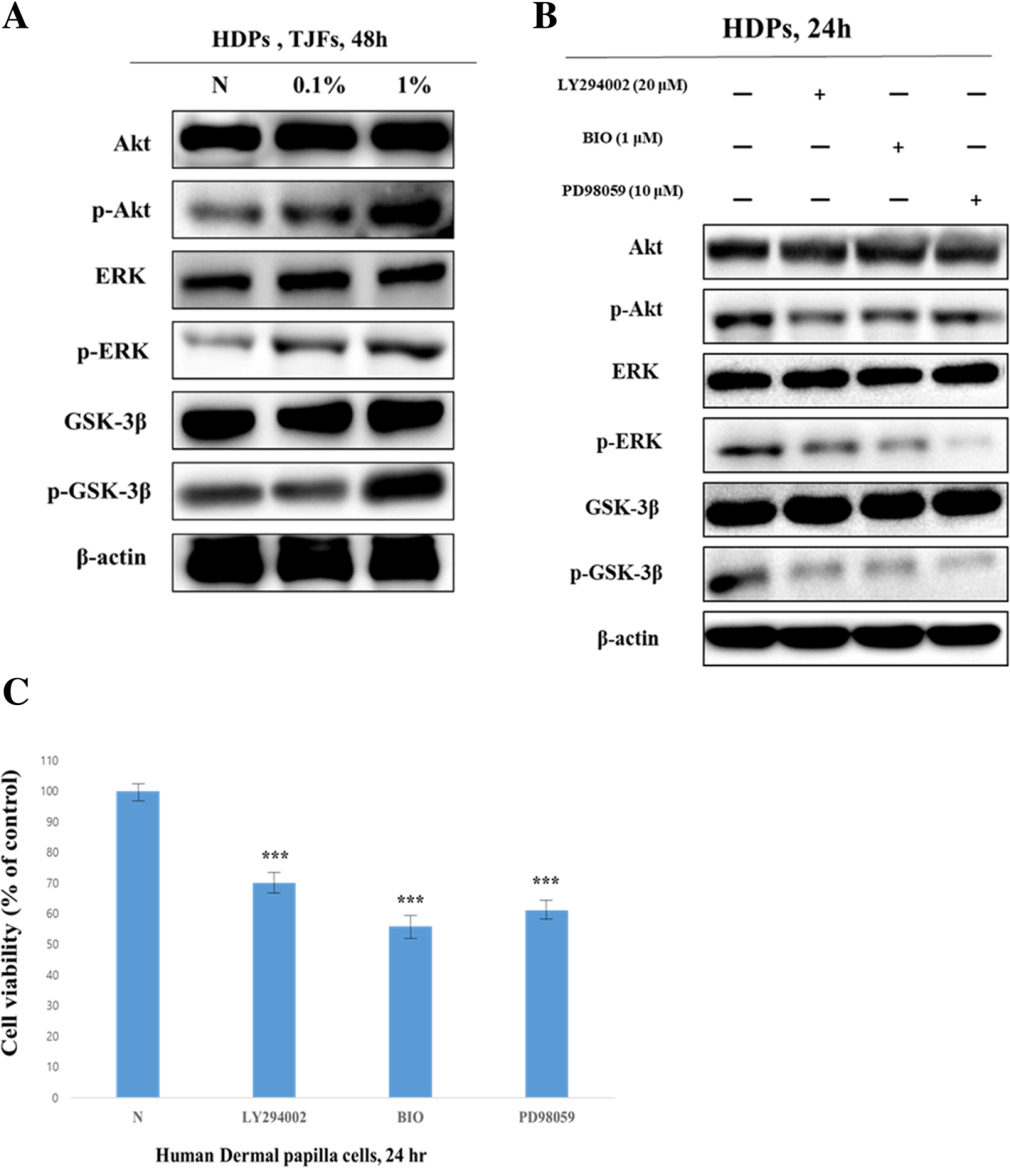
**a** TJFs stimulated HDPs proliferation through regulation of Akt/ERK/GSK-3β signaling pathway in dose-dependent manner. Protein levels were determined by Western blot analysis. The β-actin probe served as protein-loading control. **b** and **c** treatment of LY294002 or PD98059 or BIO regulates cell survive in HDPs through WST-1 assay and Western blot analysis. The statistical analysis of the data was carried out by use of an independent sample ANOVA test. ^*^p < 0.05, ^**^p < 0.01 and ^***^p < 0.001 vs. con (24 h). ^###^*p* < 0.001 (each experiment, *n* = 3). NS, not significant

To confirm the association between TJF-induced HDP cell proliferation and the Akt/ERK/GSK-3β signaling pathway, we performed a western blot to examine the Akt/ERK/GSK-3β protein levels. The expression of p-Akt, p-ERK, and p-GSK-3β increased dose-dependently following TJF treatment (Fig. 6a). The phosphorylated expression increased after TJF treatment at 0.1 and 1% compared with the control. Furthermore, to verify that the Akt/ERK/GSK-3β pathway affected HDP proliferation, HDP cells were treated with LY294002 (an Akt inhibitor), BIO (a GSK-3β inhibitor), and PD98059 (an ERK inhibitor). The expression of p-Akt, p-ERK, and p-GSK-3β decreased following treatment with their respective inhibitors. As the phosphorylated expression decreased, HDP proliferation was significantly inhibited (Fig. 6b and c). Thus, these data demonstrated that TJFs induced HDP proliferation via an Akt/ERK/GSK-3β signaling-dependent pathway.

### TJF treatment association with hair growth activity in an ex vivo model

**Fig. 7:**
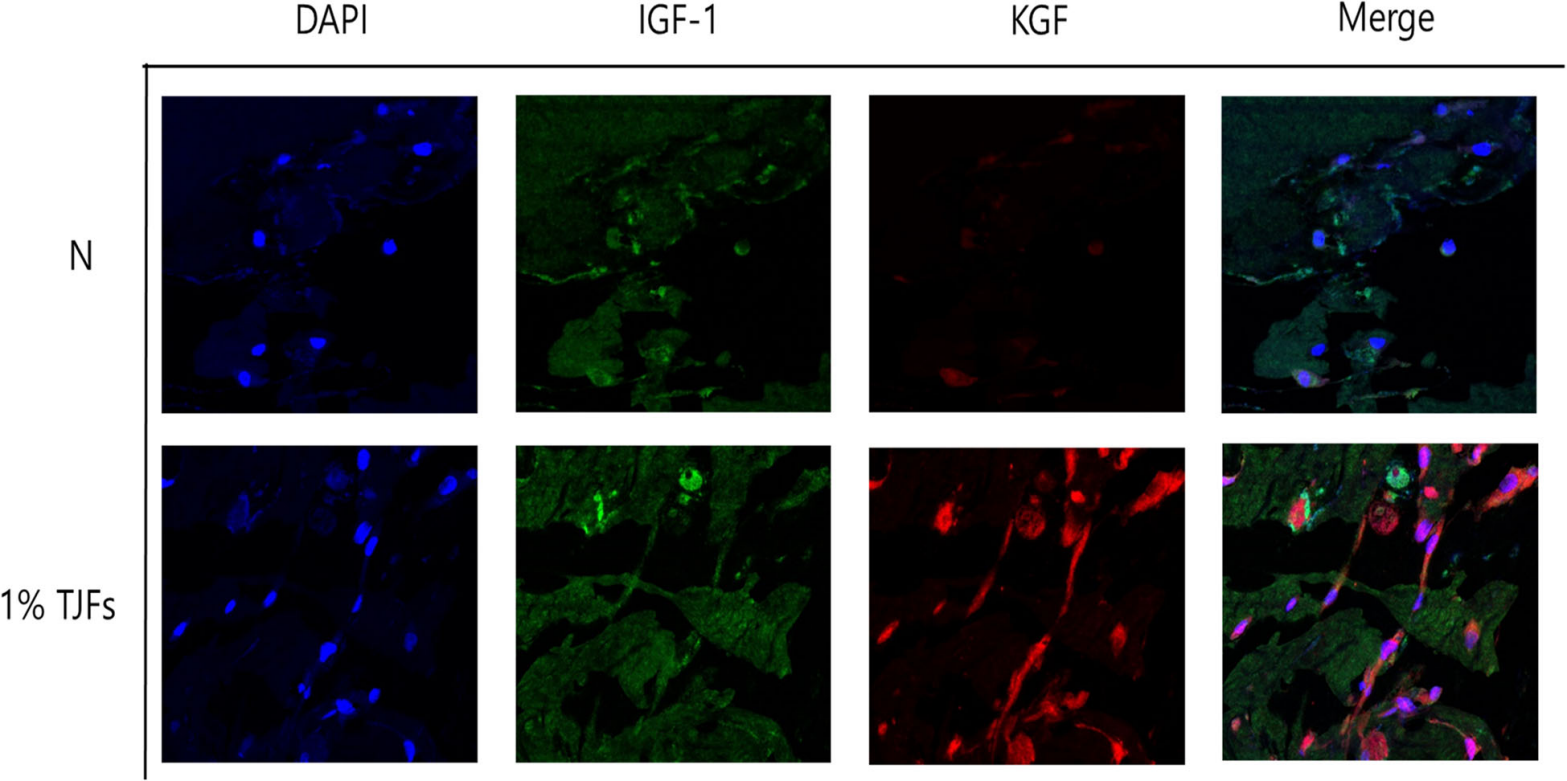
Fluorescence IHC ex vivo. The Organoytpic three-dimensional models using HDPs were immunostained for DAPI, IGF-1 and KGF. The sections were represented by a confocal microscope (Olympus, Japan)

We investigated whether HDP cells activated by TJFs possessed hair growth activity by treating an ex vivo model with TJFs for 14 days. Then, KGF and IGF-1 expression were detected immunohistochemically (Fig. 7). Our results revealed that KGF expression significantly increased, whereas IGF-1 was not significantly increased following HDP cell treatment with TJFs. This confirmed that TJF was associated with hair growth by promoting KGF expression.

## Discussion

The results from this study have demonstrated that the treatment of HDP cells with TJFs promoted hair growth activity, cell cycle progression, and vascular expansion (related to VEGF). Moreover, it inhibited apoptosis and type I 5α-reductase activity. Unlike natural extracts, fermentation by two species of microbes increased the production of peptides and beneficial components. We isolated useful peptides from the TJFs using various solvents. These peptides caused HDP proliferation. Using an *in ovo* CAM assay, we discovered that the TJFs induced angiogenesis effects through the vitamin C treatment group. Additionally, we confirmed that the activity of type I 5α-reductase, one of the proteins responsible for hair loss, was suppressed by TJFs. Apoptosis is triggered during the resting phase, causing hair loss. Our data revealed that TJFs inhibited apoptosis in HDP cells by decreasing caspase 3 activation. When HDP cells were treated with TJFs, p-Akt, p-ERK, and p-GSK-3β expression increased in a dose-dependent manner. We identified the underlying mechanism of this effect as Akt/ERK/GSK-3β signaling, confirmed by the inhibitory effects of LY294002, BIO, and PD98059 on Akt, GSK-3β, and ERK expression, respectively. Moreover, cylin E expression increased and p-CDK2 decreased following TJF treatment of HDP cells. These results suggested that TJFs affect the proliferation and cell cycle progression of HDP cells via the Akt/ERK/GSK-3β pathway, inhibit type I 5α-reductase activity, and prevent apoptosis. Finally, we determined that IGF-1 and KGF are biomarkers of hair growth in the hair bulb. By means of a three-dimensional cell culture model that mimicked the environment of actual hair follicles using HDP cells, IGF-1 and KGF protein expression confirmed that TJFs stimulate HDP cells to induce hair growth and inhibit hair loss. Previous studies have suggested that IGF-1 is essential for growth in the anagen phase and inhibits apoptosis during the catagen phase of the hair cycle [33]. Furthermore, KGF is most beneficial for stimulating hair growth factors, including VEGF, and inhibitors of type I 5α-reductase [34]. Future investigations should identify and isolate those substances in the TJFs that could treat alopecia. Experiments to isolate the effective substances from TJFs are ongoing in our laboratory. These substances will possess considerable potential as hair growth promoters.

## Conclusions

In conclusion, our data reveals that TJFs promote Human hair follicle dermal papilla cell proliferation in the in vitro. In vitro situations via regulation of Akt/ERK/GSK-3β signaling pathway. Also, ex vivo situations reveals that hair growth activity through a three-dimensional cell culture model.

## Abbreviations

Akt: Protein kinase B
CAM assay: Chicken Chorioallantoic Membrane assay
CH2Cl2: Methylene chloride
DMSO: Dissolved in dimethyl sulfoxide
ERK: The extracellular-signal-regulated kinase
EtOAc: Ethyl acetate
FBS: Fetal bovine serum
GSK-3β: Glycogen synthase kinase 3 beta
HDPs: Human hair Follicle dermal papilla cells
HPLC: High-performance liquid chromatography
IF: Immunofluorescence
IGF-1: Insulin-like growth factor-1
IHC: Immunohistochemistry
KGF: Keratinocyte growth factor
TJFs: *Bacillus/Trapa japonica Fruit* Extract Ferment Filtrate
VEGF: Vascular endothelial growth factor
WST-1: Tetrazolium salts
